# Love-related changes in the brain: a resting-state functional magnetic resonance imaging study

**DOI:** 10.3389/fnhum.2015.00071

**Published:** 2015-02-13

**Authors:** Hongwen Song, Zhiling Zou, Juan Kou, Yang Liu, Lizhuang Yang, Anna Zilverstand, Federico d’Oleire Uquillas, Xiaochu Zhang

**Affiliations:** ^1^Faculty of Psychology, Southwest University, ChongqingChina; ^2^CAS Key Laboratory of Brain Function & Disease, School of Life Sciences, University of Science and Technology of China, AnhuiChina; ^3^Icahn School of Medicine at Mount Sinai, New York, NYUSA; ^4^CAS Center of Medical Physics and Technology, University of Science and Technology of China, AnhuiChina; ^5^School of Humanities and Social Science, University of Science and Technology of China, AnhuiChina

**Keywords:** romantic love, resting state fMRI (rsfMRI), regional homogeneity (ReHo), functional connectivity (FC), dorsal anterior cingulate cortex (dACC), nucleus accumbens, temporo-parietal junction (TPJ), posterior cingulate cortex (PCC)

## Abstract

Romantic love is a motivational state associated with a desire to enter or maintain a close relationship with a specific other person. Functional magnetic resonance imaging (fMRI) studies have found activation increases in brain regions involved in the processing of reward, motivation and emotion regulation, when romantic lovers view photographs of their partners. However, not much is known about whether romantic love affects the brain’s functional architecture during rest. In the present study, resting state functional magnetic resonance imaging (rsfMRI) data was collected to compare the regional homogeneity (ReHo) and functional connectivity (FC) across an “in-love” group (LG, *N* = 34, currently intensely in love), an “ended-love” group (ELG, *N* = 34, ended romantic relationship recently), and a “single” group (SG, *N* = 32, never fallen in love). Results show that: (1) ReHo of the left dorsal anterior cingulate cortex (dACC) was significantly increased in the LG (in comparison to the ELG and the SG); (2) ReHo of the left dACC was positively correlated with length of time in love in the LG, and negatively correlated with the lovelorn duration since breakup in the ELG; (3) FC within the reward, motivation, and emotion regulation network (dACC, insula, caudate, amygdala, and nucleus accumbens) as well as FC in the social cognition network [temporo-parietal junction (TPJ), posterior cingulate cortex (PCC), medial prefrontal cortex (MPFC), inferior parietal, precuneus, and temporal lobe] was significantly increased in the LG (in comparison to the ELG and SG); (4) in most regions within both networks FC was positively correlated with the duration of love in the LG but negatively correlated with the lovelorn duration of time since breakup in the ELG. This study provides first empirical evidence of love-related alterations in brain functional architecture. Furthermore, the results shed light on the underlying neural mechanisms of romantic love, and demonstrate the possibility of applying a resting-state fMRI approach for investigating romantic love.

## INTRODUCTION

Romantic love, a very old topic, has been recorded in the poetry, songs, stories, myths, and legends of human civilization for 1000s of years (Jankowiak and Fischer, 1992; Baumeister et al., 1993). It has been regarded as the inspiration for some of the most extraordinary achievements of mankind (Bartels and Zeki, 2000), and plays an important role in human survival, reproduction, development, and evolution (Fisher, 1998).

Within the last century, romantic love has also become a topic of interest for scientists. Psychologists, for example, define romantic love as a motivational state associated with a desire to enter or maintain a close relationship with a specific other person (Aron and Aron, 1991; Cacioppo et al., 2012; Diamond and Dickenson, 2012). Love has also been shown to play a role in mediating reward and goal-directed motivation (Cacioppo et al., 2012; Diamond and Dickenson, 2012). It can alter cognition and behavior, such as promoting intensely focused attention on the preferred individual, accompanied by euphoria, craving, obsession, compulsion, distortion of reality, emotional dependence, personality changes, and risk-taking (Peele and Brodsky, 1975; Clark and Mills, 1979). Romantic love is thus a complex sentiment, involving emotional, cognitive, and behavioral components (Sternberg, 1986; Hazan and Shaver, 1987).

In recent years, researchers have devoted increasing attention to the neurobiological substrates and neurological processes of romantic love. Bartels and Zeki (2000) published the first functional magnetic resonance imaging (fMRI) study investigating the brain of a person looking at a photograph of someone whom they love. Many other researchers have further studied the pattern of brain activity of those who are in love using similar tasks (Bartels and Zeki, 2000, 2004; Aron et al., 2005; Ortigue et al., 2007; Acevedo and Aron, 2009; Fisher et al., 2010; Xu et al., 2011). Reviews of these studies conclude that love is accompanied by significantly increased activation in brain regions such as the ventral tegmental area (VTA), medial insula, anterior cingulate cortex (ACC), hippocampus, nucleus accumbens (NAC), caudate nucleus, and hypothalamus. At the same time, deactivations can be found in the amygdala, prefrontal cortex (PFC), temporal poles, and temporo-parietal junction (TPJ; Zeki, 2007; de Boer et al., 2012; Diamond and Dickenson, 2012; Tarlaci, 2012). Cacioppo et al. (2012) have suggested that romantic love-related brain regions can be divided into subcortical and cortical brain networks where the former mediates reward, motivation, and emotion regulation, and the latter mainly supports social cognition, attention, memory, mental associations, and self-representation.

However, it remains unclear whether romantic love also affects the functional architecture of the brain. After Biswal et al. (1995) proposed that functional connectivity (FC) can be studied using resting state functional magnetic resonance imaging (rsfMRI), Raichle et al. (2001) proposed the use of rsfMRI for investigating the brain when no specific task is pursued. Compared to task-fMRI, rsfMRI is a tool for exploring the intrinsic functional architecture of the brain (Fox and Raichle, 2007; Van Den Heuvel and Hulshoff Pol, 2010; Chou et al., 2012; Lee et al., 2013). This approach helps avoid potential confounds and limitations encountered in task-based approaches (e.g., practice, ceiling or floor effects, or differential performance levels; Di Martino et al., 2008). RsfMRI thus provides promising opportunities for investigating the functional topology of the brain and has been widely used to study differences between populations, too (Fox and Raichle, 2007; Van Den Heuvel and Hulshoff Pol, 2010).

Most rsfMRI studies have adopted FC to examine the correlations and dynamics between brain networks. FC is defined as the correlation of spontaneous blood oxygen level-dependent (BOLD) signals between spatially remote regions (Aertsen et al., 1989; Friston et al., 1993). This measure describes the relationship between neuronal activation patterns of anatomically separated brain regions and networks (Van Den Heuvel and Hulshoff Pol, 2010). FC has been widely used to study clinical populations such as schizophrenia (Lynall et al., 2010), Parkinson’s disease (Stoffers et al., 2008), autism spectrum disorder (Koshino et al., 2008), depression (Greicius et al., 2007), and substance abuse and dependence (Liu et al., 2010). However, FC provides little information about local features of spontaneous brain activity observed in individual regions.

In contrast, Regional Homogeneity (ReHo) is a local measurement of FC, defined as the temporary similarity between a given voxel and its neighbors (Zang et al., 2004). In this method, Kendall’s coefficient of concordance (KCC) (Zang et al., 2004) is used to measure the correlation between the time series of a given voxel and its nearest neighbor voxels in a voxel-wise way. ReHo is a validated measure of brain functioning, measuring the synchronized oscillatory activity in the cerebral cortex that is essential for spatiotemporal coordination and integration of activity in anatomically distributed but functionally related neural elements (Van Rooy et al., 2005). Neuronal synchronization is also hypothesized to underlie the efficient organization of information processing in the brain (Buzsáki and Draguhn, 2004), facilitating the coordination and organization of information processing across several spatial and temporal ranges (Fox et al., 2005). In the past years, ReHo has been used to study a variety of populations including patients suffering from schizophrenia (Liu et al., 2006), Parkinson’s disease (Wu et al., 2009), autism spectrum disorder (Paakki et al., 2010; Shukla et al., 2010), and depression (Yao et al., 2009).

Given that romantic love is a motivational state (Aron and Aron, 1991; Cacioppo et al., 2012; Diamond and Dickenson, 2012) and that there are many specific psychological and behavioral changes in romantic lovers (such as intensely focused attention on a preferred individual, obsession, and risk-taking; Peele and Brodsky, 1975; Clark and Mills, 1979) as well as facilitation of cognitive behavior (Bianchi-Demicheli et al., 2006; Ortigue et al., 2007), it is not strange to assume that being in love may affect the underlying functional architecture structure of the involved brain regions. In the present study, we computed both ReHo and FC from rsfMRI data to investigate these proposed alterations in functional brain architecture in romantic lovers.

## MATERIALS AND METHODS

### ETHICS STATEMENT

This study was approved by the Ethics Committee of Southwest University. Written informed consent was obtained from all participants. All participants were informed that their participation was completely voluntary and that they may withdraw themselves at any time. All participants were over 18 years of age.

### PARTICIPANTS

One hundred healthy college students were enrolled in the study. All participants were recruited from Southwest University (SWU, Chongqing, China) by flyers and Internet advertisement. They were interviewed at the beginning of the study procedure regarding previous romantic relationships and demographic characteristics. The participants were divided into three groups according to their previous romantic relationship: (1) the “in-love” group (LG; *N* = 34), consisting of individuals currently intensely in love; (2) the “ended-love” group (ELG; *N* = 34), consisting of individuals who had recently ended a close romantic relationship and were not currently in love; and (3) the “single” group (SG; *N* = 32), consisting of individuals who had never fallen in love with anyone.

There were no significant differences in family income, personal monthly expenses, age, or years of education (*P* > 0.1) among either of the three groups (**Table 1**). The length of time in love of participants in the LG was between 4 and 18 months (12.21 ± 3.33). In the ELG, duration since the last romantic relationship breakup was between 2 and 17 months (10.41 ± 2.97), while the length of relationship before breaking-up was 4–39 months (15.12 ± 9.91). All participants were of heterosexual orientation.

**Table 1:** Economic status, demographic, and romantic relationship status of participants.

	LG (In-love group) (*N* = 34, 16 females)		SG (Single group) (*N* = 32, 14 females)		ELG (Ended-love group) (*N* = 34, 15 females)		*F*	*P*
	Mean	SD	Mean	SD	Mean	SD		
Family income (RMB/months)	4.10 × 10^3^	7.04 ×10^2^	4.04 × 10^3^	6.22 × 10^2^	4.04 × 10^3^	6.82 × 10^2^	0.1	0.91
Monthly expenses (months)	7.35 × 10^2^	78.32	7.41 × 10^2^	86.7	7.40 × 10^2^	63.72	0.05	0.95
Age (years)	21.23	2.45	21.4	1.9	21.15	2.1	0.13	0.88
Years of education (years)	13.23	2.45	13.4	1.92	13.15	2.1	0.12	0.89
Intensity of the love (PLS scores)	104.21	10.58						
Length of time in-love (months)	12.21	3.33			12.11	9.91		
Lovelorn duration since breakup of romantic relationship (months)					10.41	2.97		

LG, participants who were intensely in love; SG, participants who had never fallen in love with someone; ELG, participants who had just ended a romantic relationship recently and were not currently in-love. PLS, Passionate Love Scale.

### SELF-RATED QUESTIONNAIRES

The Passionate Love Scale [PLS; Hatfield and Sprecher, 1986] was used to measure the status of passionate/romantic love in the LG. The PLS has been previously used in a sample of Chinese college students (Xu et al., 2011; Yin et al., 2013). Average PLS score in the LG was 104.21, and SD was 10.58.

### SCANNING ACQUISITION AND IMAGE PREPROCESSING

All imaging data were acquired using a 3T Siemens scanner (Siemens Medical, Erlangen, Germany) at the Brain Imaging Research Center of Southwest University. Resting state fMRI (rsfMRI) data were acquired using a T2∗-weighted echo-planar imaging sequence [time repetition (TR) = 2000 ms; time echo (TE) = 30 ms; flip angle = 90^∘^; field of view (FOV) = 220 mm; Matrix = 64 × 64, 32 slices; 3 mm slice thickness; voxel size = 3.4 mm × 3.4 mm × 3 mm]. For each participant 242 contiguous EPI functional volumes were collected during one run of 8 min and 4 s. Participants were instructed to lie in the scanner with eyes closed while thinking of nothing, and remaining still, relaxed, and awake throughout the scanning session (Hao et al., 2013). Additionally, high-resolution T1-weighted spin-echo images were collected (TR/TE = 1900 ms/2.52 ms; flip angle = 9^∘^; FOV = 256 mm; Matrix = 256 × 256; 1 mm slice thickness, 176 slices; voxel size = 1 mm × 1 mm × 1mm).

Imaging data were analyzed by Statistical Parametric Mapping software (SPM8; http://www.fil.ion.ucl.ac.uk/spm/software/spm8/) using two processing toolkits [the Data Processing Assistant for Resting-State fMRI (DPARSF; Chao-Gan and Yu-Feng, 2009); and the resting-state fMRI data analysis Toolkit (REST; Song et al., 2010)]. Prior to processing, the first five functional volumes of each session were discarded to allow for scanner stabilization. Cerebrospinal fluid (CSF), and the white matter signals were removed by classifying them as nuisance variables so as to reduce the effect of head motion and non-neural BOLD fluctuations (Fox et al., 2005; Kelly et al., 2008). In the present study, we used white matter (white.nii), CSF (csf.nii), and the whole brain activity signal to perform a matrix multiplication to obtain the signal of the white matter and CSF (Chao-Gan and Yu-Feng, 2009). Data preprocessing using DPARSF consisted of: (1) slice-timing correction using Fourier interpolation to correct for differences in slice acquisition time; (2) 3D motion correction using least-squares alignment and a 3 translational and 3 rotational parameter linear transformation to correct for inter-scan head motion [movement threshold for translation (*x,y,z* direction) was set at 2 mm; rotational movement (roll, pitch, yaw) threshold was set at 2^∘^]; (3) spatial normalization to a standard template (Montreal Neurological Institute) with resampling to 3 mm × 3 mm × 3 mm; (4) spatial smoothing using a 4-mm full-width-at-half-maximum (FWHM) Gaussian kernel; and (5) temporal band-pass filtering (0.01–0.08 Hz) to reduce low-frequency drift and high-frequency physiological noise.

### DEFINITION OF SEED REGIONS

Based on previous results in task-fMRI studies of romantic love (Bartels and Zeki, 2000, 2004; Aron et al., 2005; Ortigue et al., 2007; Fisher et al., 2010; Xu et al., 2011), we selected ten regions of interest (ROIs) as seed regions for the FC analysis. Each ROI was a small 10 mm centered sphere (**Table 2**). To ensure that each ROI included only voxels of one brain region, these spheres were additionally masked with a corresponding region-mask to exclude neighboring anatomical structures.

**Table 2:** Seed regions of interest (ROIs) and their MNI coordinates.

ROI (radius, 10 mm)	Left			Right			Reference
	x	y	z	x	y	z	
dACC	-6	18	32	6	33	23	Bartels and Zeki (2000, 2004), Aron et al. (2005), Fisher et al. (2010)
Caudate	-12	6	15	15	9	21	Bartels and Zeki (2000, 2004), Aron et al. (2005), Ortigue et al. (2007), Xu et al. (2011), Acevedo et al. (2012)
Insula	-33	15	-15	30	18	-15	Bartels and Zeki (2004), Ortigue et al. (2007), Fisher et al. (2010), Acevedo et al. (2012)
TPJ	-57	-42	18	51	-39	24	Bartels and Zeki (2000, 2004), Ortigue et al. (2007, 2010)
PCC	-6	-45	21	8	-44	24	Bartels and Zeki (2000, 2004), Aron et al. (2005), Ortigue et al. (2007), Fisher et al. (2010), Acevedo et al. (2012)

### FUNCTIONAL CONNECTIVITY (FC) ANALYSIS

The correlation maps for these seed regions were produced by computing correlation coefficients between the mean time series of each ROI and the time series of all other brain voxels for each participant. Correlation coefficients were converted to *z*-values using Fisher’s *r*-to-*z* transform to improve normality. In order to compare the FC across the three groups, a one-way Analysis of Variance (ANOVA) was calculated for each ROI based on the individual maps. Group analyses were thresholded using false discovery rate (FDR) correction (*P* < 0.05).

### ReHo ANALYSIS

Following procedures from a previous study (Chao-Gan and Yu-Feng, 2009), a whole brain map of ReHo values was calculated, voxel-wise, for each participant before spatial smoothing. In order to reduce the effects of variability across participants, the ReHo value of each voxel was normalized by dividing it by the mean whole-brain ReHo value of each participant (Chao-Gan and Yu-Feng, 2009; Wu et al., 2009; Liu et al., 2011; Zhang et al., 2012). The individual ReHo maps were compared across the three groups by using a ANOVA with FDR correction (*P* < 0.05).

### CORRELATION ANALYSIS BETWEEN rsfMRI AND BEHAVIOR

To investigate brain–behavior relationships we conducted simple regression analyses, regressing either ReHo/FC on the length of time in love (in the LG) or the lovelorn duration since breakup (in the ELG). Individual ReHo *z*-values were extracted from small ROI spheres (6 mm radius) placed where we found differences in the previous ReHo analyses across the three groups. The individual FC *z*-values were extracted from ROIs based on results from the comparison of FC across groups (FDR, *P* < 0.05).

## RESULTS

### REGIONAL HOMOGENEITY (ReHo) DIFFERENCES BETWEEN GROUPS

**FIGURE 1:**
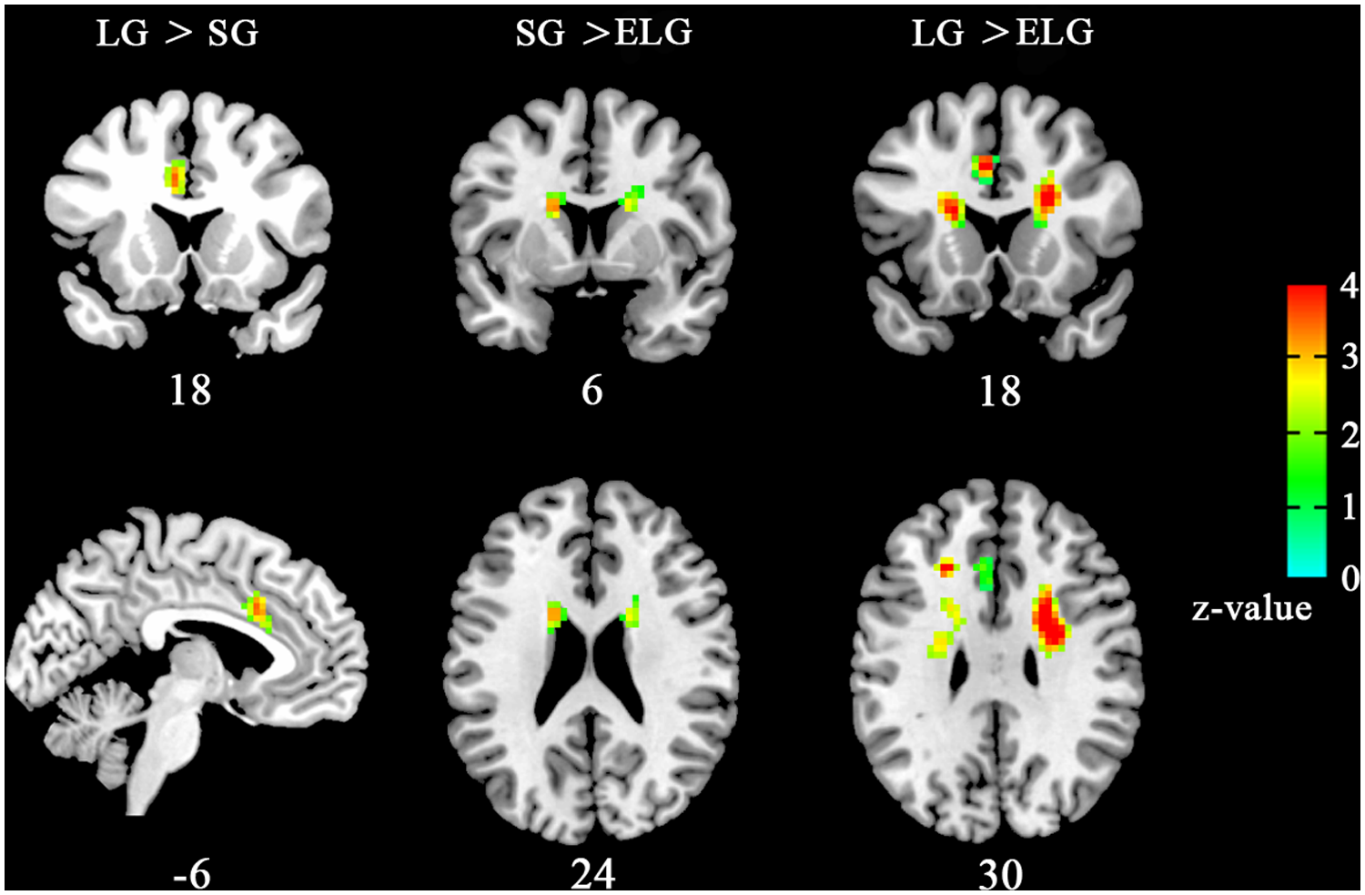
**Brain areas with altered ReHo the in-love group (LG) and ended-love group (ELG).** Significantly increased regional homogeneity (ReHo) was found in the left dorsal anterior cingulate cortex (dACC; -6,18,33) in the LG (LG > SG), but reduced ReHo was found in the left caudate nucleus [ELG < SG, (-15,9,21); ELG < LG, (-18,9,24)] and the right caudate nucleus [ELG < SG, (18,9,2); ELG < LG, (18,12,18)] in the ELG. All resultants were corrected by FDR correction (*P* < 0.05). *Coordinates in MNI space.

Results showed that ReHo was significantly increased in the LG in the left dorsal anterior cingulate cortex (dACC) [LG > SG, peak coordinates (–6,18,33); LG > ELG, peak coordinates (–6,18,30)]. Furthermore, significant reduced ReHo was found in the ELG in the left caudate nucleus [LG > ELG, peak coordinates (–15,9,21); SG > ELG peak coordinates (–18,9,24)] and right caudate nucleus [LG > ELG, peak coordinates (18,9,21); SG > ELG, peak coordinates (18,12,18)] (See **Figure 1**).

### FUNCTIONAL CONNECTIVITY (FC) DIFFERENCES BETWEEN GROUPS

**FIGURE 2:**
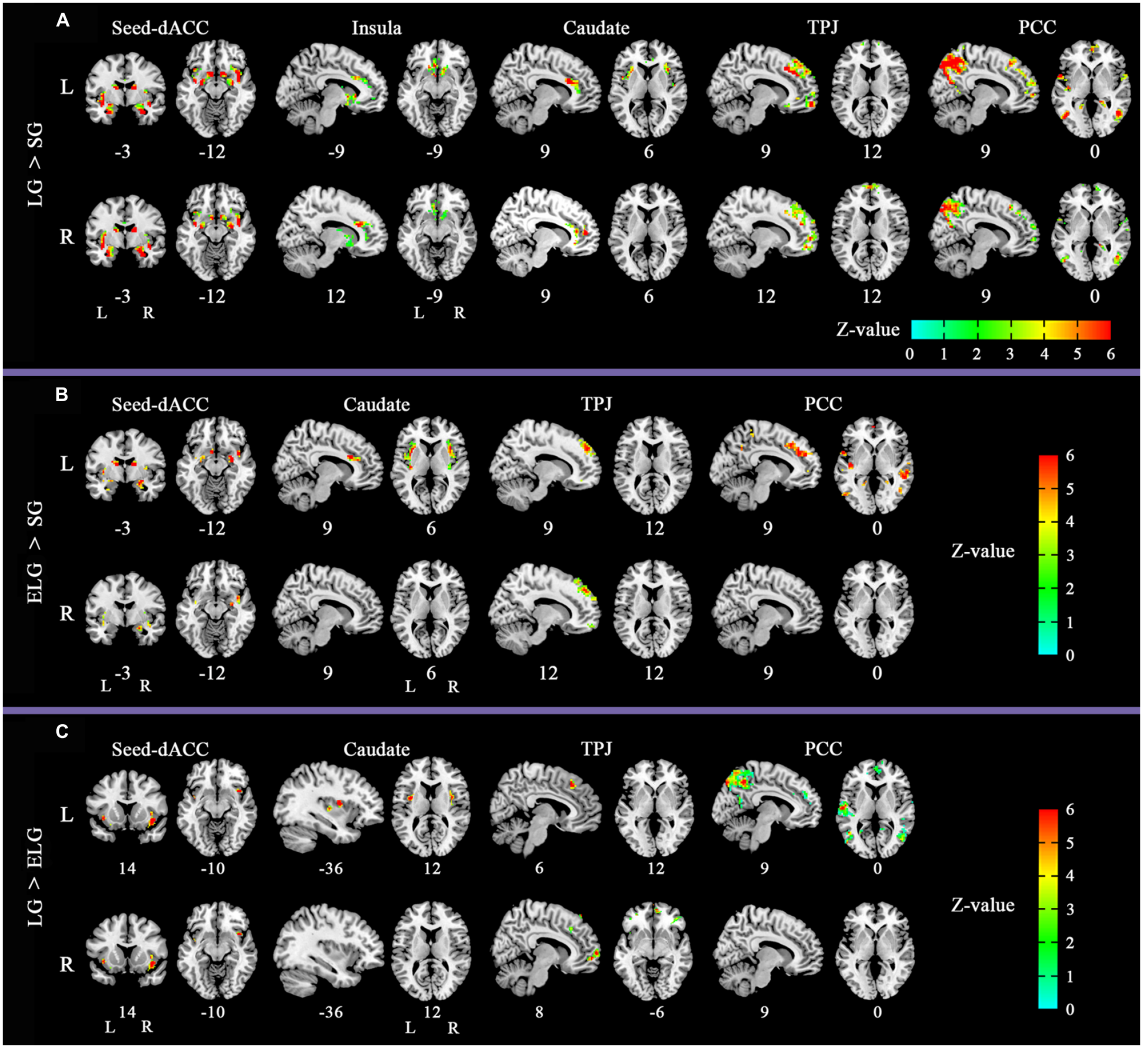
**Altered functional connectivity (FC) pattern in comparison across the three groups.** Images of FC demonstrates differences in resting-state FC between groups **(A)** LG > SG, **(B)** ELG > SG, **(C)** LG > ELG (see **Tables 3–5** for complete results). For each comparison, the top row shows the FC pattern of the left hemisphere and the bottom row shows the right hemisphere. L, left; R, right. All resultants were corrected by FDR correction (*P* < 0.05). *Coordinates in MNI space.

The between-group comparison results of FC showed that the LG (in comparison to the SG) had significantly increased FC between the dACC seed and insula, NAC, and amygdala; between the insula seed and NAC, caudate nucleus, and amygdala; between the caudate seed and dACC, and insula; between the TPJ seed and ventromedial prefrontal cortex (vMPFC), and dorsal medial prefrontal cortex (dMPFC); and between the posterior cingulate cortex (PCC) seed and the inferior parietal lobe, MPFC, precuneus, and temporal lobe (See **Figure 2**; **Table 3**).

**Table 3:** Significant regions in the comparison of functional connectivity (FC) between the LG and SG.

Seed ROI	Location of naximum intensity voxel	Cluster size (Voxels)	MNI coordinates			
			x	y	z	z-value
**LG > SG**
**Left dACC**
	Left insula	221	-36	0	0	4.93
	Right insula	305	33	12	-15	5.22
	Left NAC	28	-12	6	-12	3.67
	Left amygdala	49	-21	0	-12	3.75
	Right amygdala	51	-24	-6	-18	4.04
**Right dACC**
	Left insula	335	-36	-3	-9	6.09
	Right insula	337	39	0	-12	5.82
	Right NAC	28	9	9	-12	4.25
	Left amygdala	80	-30	-3	-21	5.33
	Right amygdala	95	-27	0	-18	4.3
**Left insula**
	Left caudate	89	-12	18	-3	3.93
	Right caudate	65	15	21	-6	4.16
	Left amygdala	80	-21	0	-12	3.79
**Right insula**
	Left NAC	20	-12	9	-12	1.44
	Right NAC	25	12	9	-9	2.51
	Left caudate	160	-12	-9	18	4.23
	Right caudate	188	18	21	-3	3.49
	Left amygdala	59	-27	-6	-18	5.34
	Right amygdala	69	30	-9	-12	4.07
**Left caudate**
	Right dACC	58	9	21	24	5.09
	Left insula	50	-45	6	3	4.25
	Right insula	73	48	9	-6	4.9
**Right caudate**
	Right dACC	34	12	27	21	3.6
**Left TPJ**
	Left vMPFC	39	-12	63	-3	4.89
	Right vMPFC	73	9	63	-12	4.21
	Left dMPFC	362	0	30	39	5.61
	Right dMPFC	305	6	30	39	5.01
**Right TPJ**
	Left vMPFC	103	0	60	-12	4.3
	Right vMPFC	93	12	54	-12	4.31
	Left dMPFC	365	-3	27	39	5.4
	Right dMPFC	192	12	45	24	4.29
**Left PCC**
	Left inferior parietal	342	-27	-48	51	5.01
	Right inferior parietal	109	30	-48	54	4.97
	Left MPFC	142	0	-3	54	3.96
	Right MPFC	377	6	27	54	4.11
	Left precuneus	560	-3	-57	66	5.57
	Right precuneus	515	9	-60	57	4.53
	Left temporal lobe	531	-45	-63	-9	5.15
	Right temporal lobe	772	48	-15	-18	5.79
**Right PCC**
	Left inferior parietal	238	-39	-57	60	4.83
	Right inferior parietal	80	30	-48	54	4.15
	Left MPFC	63	0	3	54	4.04
	Left precuneus	387	-6	-63	66	6.06
	Right precuneus	327	6	-60	51	4.27
	Left temporal lobe	254	-60	-12	9	5.4
	Right temporal lobe	270	51	-51	-21	5.19
**LG < SG**						
	None					

*NAC, nucleus accumbens; MPFC, medial prefrontal cortex; vMPFC, ventral medial prefrontal cortex; dMPFC, dorsomedial prefrontal cortex. FDR correction (*P* < 0.05) for multiple comparisons, MNI coordinates (*x,y,z*) for the most significant voxel in a cluster.*

In comparison to the ELG, the LG also showed significantly increased FC between the dACC seed and insula; between the caudate nucleus seed and insula; between the TPJ seed and vMPFC, and dMPFC; and between the PCC seed and inferior parietal lobe, MPFC, precuneus, and temporal lobe (See **Table 4**).

**Table 4:** Significant regions in the comparison of FC between the LG and ELG.

Seed ROI	Location of maximum intensity voxel	Cluster size (Voxels)	MNI coordinates			z-value
			x	y	z	
**LG > ELG**						
**Left dACC**
	Left insula	34	-39	6	0	2.81
	Right insula	78	36	18	0	3.62
**Right dACC**
	Left insula	18	-45	6	0	2.39
	Right insula	44	36	24	0	3.02
**Left caudate**
	Left insula	13	-36	0	12	3.18
	Right insula	29	39	0	15	3.49
**Left TPJ**
	Right dMPFC	189	6	27	48	3.22
**Right TPJ**
	Left vMPFC	72	9	66	6	3.15
	Left dMPFC	39	-12	18	48	2.66
	Right dMPFC	37	12	30	42	2.27
**Left PCC**
	Right inferior parietal	35	36	-48	48	2.38
	Left MPFC	47	-3	36	27	3.31
	Left precuneus	195	-12	-78	48	2.69
	Right precuneus	290	6	-60	48	3.35
	Right temporal lobe	116	54	-66	21	2.86
**LG<ELG**						
	None					

FDR correction (*P* < 0.05), MNI coordinates (*x,y,z*) for the most significant voxel in a cluster.

In comparison to the SG, the ELG showed significantly increased FC between the dACC seed and insula, amygdala, caudate nucleus, and NAC; between the caudate nucleus seed and dACC, and insula; between the TPJ seed and vMPFC, and dMPFC; between the PCC seed and inferior parietal lobe, MPFC, precuneus, and temporal lobe (See **Table 5**).

**Table 5:** Significant regions in the comparison of FC between the ELG and SG.

Seed ROI	Location of maximum intensity voxel	Cluster size (Voxels)	MNI coordinates			z-value
			x	y	z	
**ELG > SG**						
**Left dACC**
	Left insula	76	-36	-9	9	3.57
	Right insula	77	36	-24	21	4.64
	Left NAC	20	-12	9	-12	2.53
	Left amygdala	29	-24	-6	-12	3.7
	Right amygdala	41	30	-6	-12	5.41
**Right dACC**
	Left insula	226	-36	-6	-6	4.47
	Right insula	241	33	-18	-18	5.29
	Left amygdala	30	-27	-6	-15	3.46
	Right amygdala	48	27	-6	-12	3.85
**Left caudate**
	Left dACC	35	-6	21	30	3.24
	Right dACC	43	6	18	27	5.35
	Left insula	139	-39	-15	0	4.81
	Right insula	121	39	15	-3	5.26
**Left TPJ**
	Left dMPFC	152	-6	48	48	5.61
	Right dMPFC	73	12	51	39	5.28
**Right TPJ**
	Left vMPFC	32	-3	57	-15	4.26
	Left dMPFC	134	-9	57	36	4.76
	Right dMPFC	82	12	48	42	4.7
**Left PCC**
	Left inferior parietal	75	-54	-48	42	5.29
	Right inferior parietal	42	57	-42	48	4.48
	Left MPFC	280	-3	36	36	5.61
	Right MPFC	261	3	57	36	5
	Left precuneus	37	-15	-39	66	4.43
	Left temporal lobe	165	-42	-15	-3	5.38
	Right temporal lobe	421	57	-18	-15	5.37
**LG<ELG<SG**						
	None					

FDR correction (*P* < 0.05), MNI coordinates (*x,y,z*) for the most significant voxel in a cluster.

### CORRELATION BETWEEN rsfMRI AND BEHAVIOR

**FIGURE 3:**
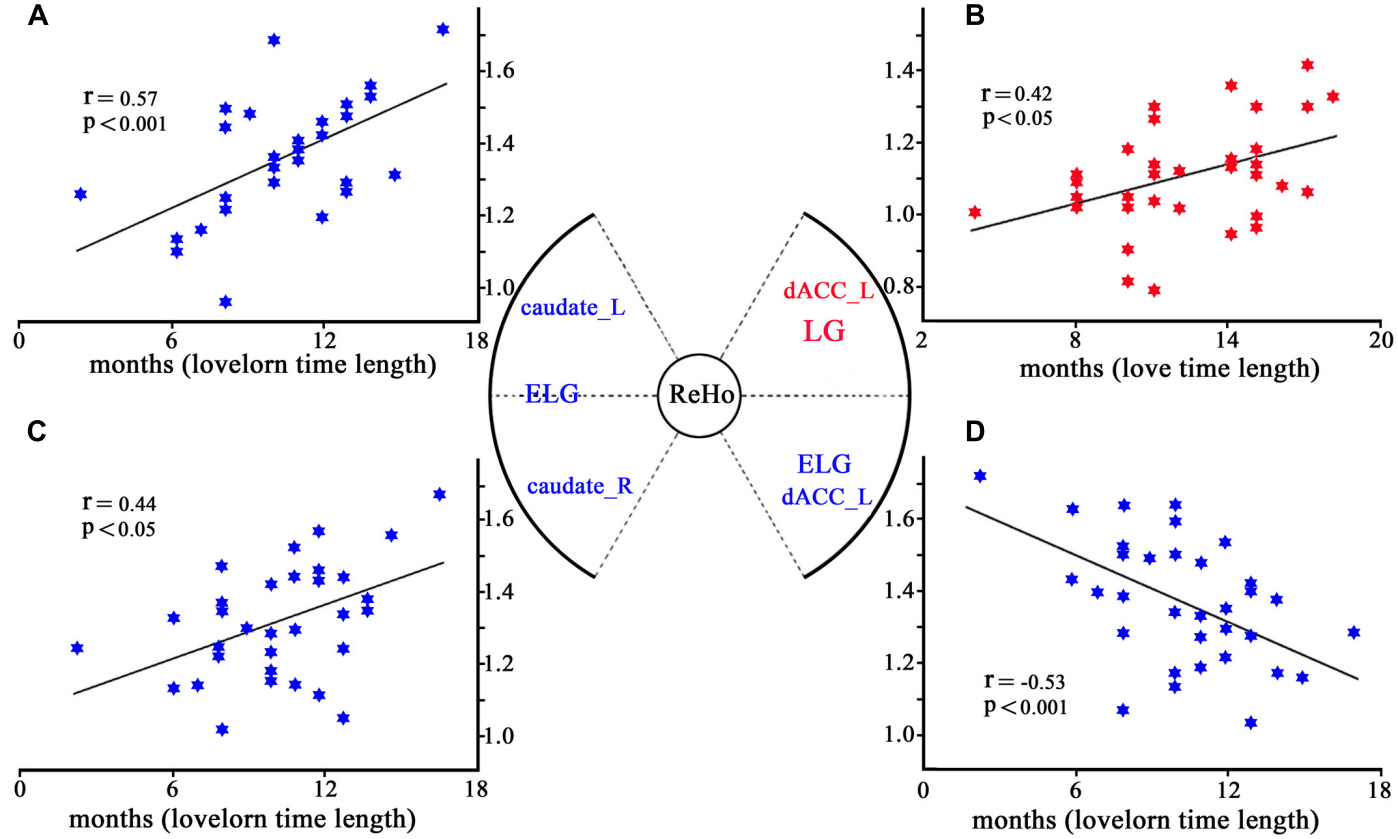
**Correlation between ReHo and the length of time in-love (in LG) or the lovelorn duration since the breakup of romantic relationship (in ELG). (A)** depicts ReHo in the left caudate nucleus (–18, 9, 24), which was significantly positively correlated with the length of time since the romantic relationship breakup in ELG; **(B)** shows ReHo in left dACC (–6,18,33), which was significantly negatively correlated with the length of time in love of LG; **(C)** demonstrates ReHo in the right caudate nucleus (18,12,18), which was significantly positively correlated with the length of time since the romantic relationship breakup in ELG; **(D)** shows ReHo of left dACC (–6,18,30), which was significantly positively correlated with the length of time since the romantic relationship breakup of ELG. *Coordinates in MNI space.

Regression analyses showed that while ReHo of the left dACC (–6,18,33) significantly increased with the length of time in love in the LG, it was significantly decreased with the lovelorn duration of time since breakup of romantic relationship in the ELG. Furthermore, while ReHo of the bilateral caudate nucleus was not correlated with the length of time in love in the LG, it was significantly positively correlated with lovelorn duration of time since breakup in the ELG (**Figure 3**).

**FIGURE 4:**
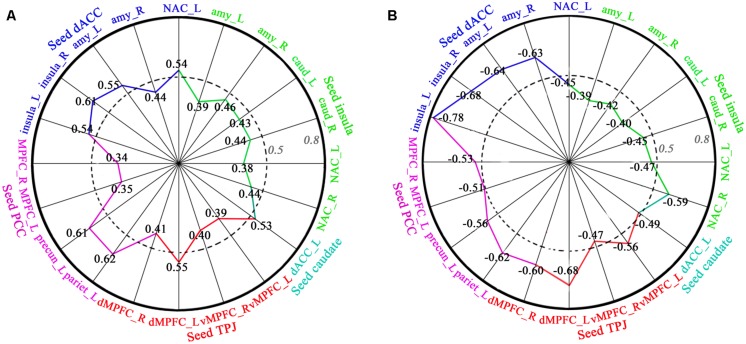
**Correlation between FC and length of time in-love (in LG) and the lovelorn duration since breakup of romantic relationship (in ELG). (A)** depicts the significant positive correlation between FC and the length of time in-love in LG; **(B)** shows the significant negative correlation between FC and the lovelorn duration since the breakup of romantic relationship in ELG. Intensity of FC was extracted from ROIs (small sphere of 6 mm radius, the center coordinates are listed in **Table 3**) based on the results of the FC comparison between the LG and SG (FDR, *P* < 0.05). The absolute value of the correlation coefficient increases gradually from the center to the circumference. Numbers represent the correlation coefficient, and the colors represent the corresponding brain regions. These are only the results of the right hemisphere. All correlations shown were significant (*P* < 0.05).

Regression analyses of FC and behavioral data showed that FC (dACC–insula, dACC–amygdala, dACC–NAC, insula–amygdala, insula–caudate, insula-NAC, TPJ–vMPFC, TPJ–dMPFC, PCC–precuneus, PCC–inferior parietal lobe, PCC–MPFC) was significantly positively correlated with length of time in love in the LG, and significantly negatively correlated with the lovelorn duration of time since breakup in the ELG (**Figure 4**).

## DISCUSSION

Although previous task-fMRI studies have preliminarily identified romantic love-related brain networks (Aron et al., 2005; Fisher et al., 2010; Xu et al., 2011), it remained unclear whether romantic love can affect the functional architecture of the brain. In the present study, we computed both ReHo and FC using rsfMRI data across three groups of participants (LG, “in-love” group who were currently intensely in love; ELG, “ended-love” group who recently ended a romantic relationship and were not currently in love; and SG, “single” group who had never fallen in love with anyone).

ReHo analysis results showed significantly increased ReHo of the left dACC in the in-love group (LG > SG, LG > ELG). Furthermore, the ReHo of the left dACC was positively correlated with the length of time in love in the LG, and was negatively correlated with lovelorn duration in the ELG, suggesting that the ReHo of the left dACC may be closely related to the state of falling in love. At the same time, the ReHo of the bilateral caudate nucleus was significantly decreased in ELG (ELG < SG, ELG < LG), and was positively correlated with lovelorn duration in the ELG, suggesting that ReHo of the caudate nucleus may be closely related to the effects of ending a love relationship.

Results of FC showed that the lover group had significantly increased FC (LG > SG, LG > ELG) within the reward, motivation, and emotion regulation brain network (including the dACC, caudate nucleus, NAC, and insula) as well as in the social cognition network (including the TPJ, PCC, mPFC, precuneus, and inferior parietal lobe). Comparable to the ReHo analysis results (in the left dACC), FCs in both networks were significantly positively correlated with the length of time in love in the LG, as well as negatively correlated with lovelorn duration in the ELG, suggesting that falling in love may also be associated with increased connectivity within certain brain networks.

### ROMANTIC LOVE AND THE REWARD, MOTIVATION AND EMOTION REGULATION NETWORK

The ACC, caudate nucleus, amygdala, NAC, and insula are core components of the brain systems that play an important role in the processing of sensory and emotional information, reward, and motivational processes (Mogenson et al., 1980). In the present study, we found significant increased FC in the LG (LG > SG, LG > ELG) between the ACC, caudate nucleus, amygdala, NAC, and insula. This may imply that romantic love may change the function of the reward, motivation, and emotion regulation brain network.

The dACC plays a key role in monitoring conflict through information processing, and compensatory adjustments in cognitive control (Botvinick et al., 2004). In fact, some researchers have found increased activation in the ACC individuals with greater social insight and maturity (Lane et al., 1998; Bush et al., 1999). Bartels and Zeki (2000) suggested that the dACC is implicated in states of happiness, interoception (i.e., attention to one’s own emotional state), and also in social interactions that involve assessing one’s own and other people’s emotions and states of mind. For example, Aron et al. (2005) found that length of time in love is positively correlated with dACC activation when watching photographs of a romantic partner.

The caudate nucleus is highly innervated by dopaminergic neurons that originate mainly from the VTA and substantia nigra pars compacta (SNc). The caudate nucleus is associated with reward detection, expectation, representation of goals, and integration of sensory inputs (Aron et al., 2005; Lauwereyns, 2006).

The amygdala is mainly responsible for processing information related to fear, sadness and aggression, and mediating emotional learning (Dalgleish, 2004). Activation level in the amygdala has been shown to decrease when participants view photos of their sweetheart (Bartels and Zeki, 2000, 2004; Aron et al., 2005; Xu et al., 2011). Furthermore, the NAC, a brain area coinciding with cortical areas rich in dopamine and oxytocin receptors, is an important part of the reward pathway that plays a central role in the visual perception of pleasant stimuli (Aharon et al., 2001; Sabatinelli et al., 2007). It is involved in both natural and abnormal reward processes (Breiter et al., 2001; Knutson et al., 2005; Baler and Volkow, 2006; Knutson and Wimmer, 2007; Cooper and Knutson, 2008). Within the context of love, the recruitment of the NAC is therefore consistent with notions of romantic love as ‘a desire for union with another’ (Hatfield and Rapson, 1993; Acevedo et al., 2012).

The insula has been ascribed a role in representing subjective feelings, attention, cognitive choices, intentions, time perception, awareness of sensations, movements (Farrer and Frith, 2002; Critchley et al., 2004; Tsakiris et al., 2007), the visual image of the self (Devue et al., 2007), subjective expectations (Seymour et al., 2004; Preuschoff et al., 2008), and the trustworthiness of other individuals (Craig, 2002). Studies of romantic love report that the activity in the insula is increased when participants view their romantic partner’s picture (Bartels and Zeki, 2004; Ortigue et al., 2007; Fisher et al., 2010).

Previous research has demonstrated that spatially remote brain regions do not function independently, but rather, interact with one another during cognitive processing. For example, when individuals engage in a reinforcement learning paradigm relating to judging the positive or negative value of visual stimuli both the amygdala and the NAC are involved in signal processing, which is then passed on to the insula (Reynolds and Zahm, 2005; Paulus and Stein, 2006). Unconditioned and conditioned sexual incentive cues are also known to be processed in the caudate nucleus, which expects, detects, and represents the reward values of the external stimulus, and outputs them to the insula (Cacioppo et al., 2012). The control of a goal-directed behavior will involve both the insula, representing awareness, and the ACC, representing the control of directed effort (Craig, 2009). Thus, increased FC between these regions in a group of lovers may be the result of frequent efforts to monitor their own emotional state, as well as their lovers’ emotional state, monitoring conflicts while adjusting cognitive strategies in order to resolve conflicts so as to maintain their romantic relationship.

### ROMANTIC LOVE AND THE SOCIAL COGNITION NETWORK

Our findings show that the LG had significantly increased FC compared to the SG and ELG between the TPJ seed and vMPFC, and dMPFC; and between the PCC seed and inferior parietal, MPFC, precuneus, and temporal lobe. Moreover, FC was significantly positively related to the length of time in love in the LG. These regions are part of a social cognition network, which contains brain areas activated during social interaction and areas involved in general cognition and attention. Regions activated during social interaction include the TPJ, vMPFC, and dMPFC. This network has been consistently associated with social, moral and ‘theory of mind’ tasks (the ability to determine other people’s emotions and intentions) (Frith and Frith, 1999; Brunet et al., 2000; Gallagher and Frith, 2003), and has been associated with social trustworthiness (Winston et al., 2002), facial expressions (Winston et al., 2002), moral judgment (Greene and Haidt, 2002; Moll et al., 2002), and attention to one’s own emotions (Lane et al., 1997; Gusnard et al., 2001). Brain regions generally involved in social cognition include the PCC and inferior parietal and middle temporal cortices, which play a role in cognitive attention, and short-and long-term memory (Beauregard et al., 1998; Maddock, 1999; Cabeza and Nyberg, 2000; Buckner et al., 2008).

### DOPAMINE, OXYTOCIN, VASOPRESSIN, AND ROMANTIC LOVE

Our results show increased FC between subcortical regions in lovers (between the caudate nucleus, NAC, amygdala, and insula), areas closely related to the mesolimbic dopaminergic system. The mesolimbic dopaminergic system is suggested to be a mechanism by which humans and other mammals enact behaviors that maintain and protect their pair-bonds (Winslow et al., 1993; Sue Carter et al., 1995; Wang et al., 1997; Aragona et al., 2003). Dopamine has also been shown to play an important role in the romantic love of humans (Acevedo et al., 2012).

The VTA is centrally placed in a wider motivational/reward network associated with behaviors necessary for survival (Camara et al., 2009). It is considered a central platform for pleasurable feelings and pair-bonding (Ortigue et al., 2010). The NAC has been implicated in the interaction between the neurotransmitter dopamine and the neuropeptide oxytocin (Liu and Wang, 2003). Both oxytocin and vasopressin have been shown to be crucially involved in romantic love and bonding (Kendrick, 2000; Fisher et al., 2006; Gonzaga et al., 2006). Oxytocin is released during sexual activity and mating, and may be the neurochemical mechanism for the anxiolytic effect of mating (Waldherr and Neumann, 2007). Recently, Rilling et al. (2012) suggested that both oxytocin and vasopressin were associated with increased FC between amygdala and the anterior insula, possibly enhancing the amygdala’s ability to elicit visceralsomatic markers in order to guide decision-making. The increased FC observed between subcortical regions in lovers may therefore reflect the neurophysiological interaction between oxytocin, dopamine, and/or vasopressin while in a state of love.

### EFFECT OF LOVELORN STATE ON BRAIN NETWORKS

Although we did not intentionally investigate the effect of lovelorn in the present study, we found that ReHo of the bilateral caudate nucleus was significantly decreased in the ELG (ELG < SG, ELG < LG) and was also correlated with the lovelorn duration of time since breakup of romantic relationship in the ELG (not correlated with the length of time in love in the LG).

As discussed before, the caudate nucleus is associated with detection of reward, expectation, representation of goals, and integration of sensory input (Aron et al., 2005; Lauwereyns, 2006). Deep brain stimulation of the caudate nucleus has been shown to improve symptoms of anxiety disorder and major depression (Aouizerate et al., 2004). Neurochemical studies have demonstrated that these effects may be mediated by non-selective corticotropic-releasing systems. Being in a relationship has been associated with elevated CRF mRNA in the bed nucleus of the stria terminalis in nerve fibers originating from the amygdal (Bosch et al., 2008). Therefore, the caudate nucleus may be very important for relieving symptoms of anxiety and depression. An elevated FC between regions involved in the anxiety-relief system after breaking up may be a sign of recovery.

### LIMITATIONS

The chosen approach was a cross-sectional design, conducted via a comparison across three independent subject groups. Further longitudinal studies will be necessary to verify and extend the findings of the present study. One challenge for longitudinal studies of romantic love may be that romantic relationships are not easily controlled inside a laboratory. Another possible limitation of this study is that we do not know exactly whether love-related alterations are adaptation, or maladaptation in lovers. From an evolutionary perspective, romantic love can be seen as a mechanism developed for choosing a partner that offers the best chances for survival to the offspring (de Boer et al., 2012). We therefore propose that love-related alterations in FC or ReHo reflect this mechanism, as it is a correlate of the individuals’ effort when trying to maintain an important inter-personal relationship. However, based on the present results we cannot directly test this hypothesis. In future studies, cognitive and behavioral tasks should therefore be employed to further investigate the relationship between resting brain functional alterations and love-related behaviors.

## CONCLUSION

In summary, we calculated Regional Homogeneity (ReHo) and functional connectivity (FC) using resting state functional magnetic resonance imaging (rsfMRI) data to investigate romantic love-related brain functional topological changes. We found that love-related alterations included increased ReHo of the left dACC and increased FC within the reward, motivation, and emotion regulation network, as well as the social cognition network. We also found decreased ReHo of the bilateral caudate nucleus related to the ending of a romantic relationship.

This study provides the first empirical evidence of love-related alterations in the underlying functional architecture of the brain. Findings are in agreement with results from task-dependent fMRI studies, and complement well the functional findings of task-dependent fMRI studies. These results shed light on the underlying neurophysiological mechanisms of romantic love by investigating intrinsic brain activity, and demonstrate the possibility of applying a resting state approach for investigating romantic love.

## Conflict of Interest Statement

The authors declare that the research was conducted in the absence of any commercial or financial relationships that could be construed as a potential conflict of interest.

## References

[B1] Acevedo B. P. Aron A. (2009). Does a long-term relationship kill romantic love? *Rev. Gen. Psychol*. 13 59–65 10.1037/a0014226

[B2] Acevedo B. P. Aron A. Fisher H. E. Brown L. L. (2012). Neural correlates of long-term intense romantic love. *Soc. Cogn. Affect. Neurosci.* 7 145–159 10.1093/scan/nsq092 21208991 PMC3277362

[B3] Aertsen A. M. Gerstein G. L. Habib M. K. Palm G. (1989). Dynamics of neuronal firing correlation: modulation of “effective connectivity”. *J. Neurophysiol.* 61 900–917. 2723733 10.1152/jn.1989.61.5.900

[B4] Aharon I. Etcoff N. Ariely D. Chabris C. F. O’Connor E. Breiter H. C. (2001). Beautiful faces have variable reward value: fMRI and behavioral evidence. *Neuron* 32 537–551 10.1016/S0896-6273(01)00491-3 11709163

[B5] Aouizerate B. Cuny E. Martin-Guehl C. Guehl D. Amieva H. Benazzouz A. (2004). Deep brain stimulation of the ventral caudate nucleus in the treatment of obsessive-compulsive disorder and major depression: case report. *J. Neurosurg.* 101 682–686 10.3171/jns.2004.101.4.0682 15481726

[B6] Aragona B. J. Liu Y. Curtis J. T. Stephan F. K. Wang Z. (2003). A critical role for nucleus accumbens dopamine in partner-preference formation in male prairie voles. *J. Neurosci.* 23 3483–3490. 12716957 10.1523/JNEUROSCI.23-08-03483.2003 PMC6742315

[B7] Aron A. Aron E. N. (1991). *Love and Sexuality.* Hillsdale: Lawrence Erlbaum Associates press.

[B8] Aron A. Fisher H. Mashek D. J. Strong G. Li H. Brown L. L. (2005). Reward, motivation, and emotion systems associated with early-stage intense romantic love. *J. Neurophysiol.* 94 327–337 10.1152/jn.00838.2004 15928068

[B9] Baler R. D. Volkow N. D. (2006). Drug addiction: the neurobiology of disrupted self-control. *Trends Mol. Med.* 12 559–566 10.1016/j.molmed.2006.10.005 17070107

[B10] Bartels A. Zeki S. (2000). The neural basis of romantic love. *Neuroreport* 11 3829–3834 10.1097/00001756-200011270-00046 11117499

[B11] Bartels A. Zeki S. (2004). The neural correlates of maternal and romantic love. *Neuroimage* 21 1155–1166 10.1016/j.neuroimage.2003.11.003 15006682

[B12] Baumeister R. F. Wotman S. R. Stillwell A. M. (1993). Unrequited love: on heartbreak, anger, guilt, scriptlessness, and humiliation. *J. Pers. Soc. Psychol.* 64 377–394 10.1037/0022-3514.64.3.377

[B13] Beauregard M. Leroux J.-M. Bergman S. Arzoumanian Y. Beaudoin G. Bourgouin P. (1998). The functional neuroanatomy of major depression: an fMRI study using an emotional activation paradigm. *Neuroreport* 9 3253–3258 10.1097/00001756-199810050-00022 9831460

[B14] Bianchi-Demicheli F. Grafton S. T. Ortigue S. (2006). The power of love on the human brain. *Soc. Neurosci.* 1 90–103 10.1080/17470910600976547 18633778

[B15] Biswal B. Zerrin Yetkin F. Haughton V. M. Hyde J. S. (1995). Functional connectivity in the motor cortex of resting human brain using echo-planar mri. *Magn. Reson. Med.* 34 537–541 10.1002/mrm.1910340409 8524021

[B16] Bosch O. J. Nair H. P. Ahern T. H. Neumann I. D. Young L. J. (2008). The CRF system mediates increased passive stress-coping behavior following the loss of a bonded partner in a monogamous rodent. *Neuropsychopharmacology* 34 1406–1415 10.1038/npp.2008.154 18923404 PMC2669698

[B17] Botvinick M. M. Cohen J. D. Carter C. S. (2004). Conflict monitoring and anterior cingulate cortex: an update. *Trends Cogn. Sci. (Regul. Ed.)* 8 539–546 10.1016/j.tics.2004.10.003 15556023

[B18] Breiter H. C. Aharon I. Kahneman D. Dale A. Shizgal P. (2001). Functional imaging of neural responses to expectancy and experience of monetary gains and losses. *Neuron* 30 619–639 10.1016/S0896-6273(01)00303-8 11395019

[B19] Brunet E. Sarfati Y. Hardy-Baylé M.-C. Decety J. (2000). A PET investigation of the attribution of intentions with a nonverbal task. *Neuroimage* 11 157–166 10.1006/nimg.1999.0525 10679187

[B20] Buckner R. L. Andrews-Hanna J. R. Schacter D. L. (2008). The brain’s default network. *Ann. N. Y. Acad. Sci.* 1124 1–38 10.1196/annals.1440.011 18400922

[B21] Bush G. Frazier J. A. Rauch S. L. Seidman L. J. Whalen P. J. Jenike M. A. (1999). Anterior cingulate cortex dysfunction in attention-deficit/hyperactivity disorder revealed by fMRI and the counting Stroop. *Biol. Psychiatry* 45 1542–1552 10.1016/S0006-3223(99)00083-9 10376114

[B22] Buzsáki G. Draguhn A. (2004). Neuronal oscillations in cortical networks. *Science* 304 1926–1929 10.1126/science.1099745 15218136

[B23] Cabeza R. Nyberg L. (2000). Neural bases of learning and memory: functional neuroimaging evidence. *Curr. Opin. Neurol.* 13 415–421 10.1097/00019052-200008000-00008 10970058

[B24] Cacioppo S. Bianchi-Demicheli F. Frum C. Pfaus J. G. Lewis J. W. (2012). The common neural bases between sexual desire and love: a multilevel kernel density fMRI analysis. *J. Sex. Med.* 9 1048–1054 10.1111/j.1743-6109.2012.02651.x 22353205

[B25] Camara E. Rodriguez-Fornells A. Ye Z. Münte T. F. (2009). Reward networks in the brain as captured by connectivity measures. *Front. Neurosci.* 3:350–362 10.3389/neuro.01.034.2009 20198152 PMC2796919

[B26] Chao-Gan Y. Yu-Feng Z. (2009). DPARSF: a MATLAB toolbox for “Pipeline” data analysis of resting-state fMRI. *Front. Syst. Neurosci.* 4:13 10.3389/fnsys.2010.00013 PMC2889691 20577591

[B27] Chou Y.-H. Panych L. P. Dickey C. C. Petrella J. R. Chen N.-K. (2012). Investigation of long-term reproducibility of intrinsic connectivity network mapping: a resting-state fMRI study. *Am. J. Neuroradiol.* 33 833–838 10.3174/ajnr.A2894 22268094 PMC3584561

[B28] Clark M. S. Mills J. (1979). Interpersonal attraction in exchange and communal relationships. *J. Pers. Soc. Psychol.* 37 12–24 10.1037/0022-3514.37.1.12

[B29] Cooper J. C. Knutson B. (2008). Valence and salience contribute to nucleus accumbens activation. *Neuroimage* 39 538–547 10.1016/j.neuroimage.2007.08.009 17904386 PMC2169259

[B30] Craig A. D. (2002). How do you feel? Interoception: the sense of the physiological condition of the body. *Nat. Rev. Neurosci.* 3 655–666 10.1038/nrn894 12154366

[B31] Craig A. D. (2009). How do you feel–now? The anterior insula and human awareness. *Nat. Rev. Neurosci.* 10 59–70 10.1038/nrn2555 19096369

[B32] Critchley H. D. Wiens S. Rotshtein P. Öhman A. Dolan R. J. (2004). Neural systems supporting interoceptive awareness. *Nat. Neurosci.* 7 189–195 10.1038/nn1176 14730305

[B33] Dalgleish T. (2004). The emotional brain. *Nat. Rev. Neurosci.* 5 583–589 10.1038/nrn1432 15208700

[B34] de Boer A. Van Buel E. M. Ter Horst G. J. (2012). Love is more than just a kiss: a neurobiological perspective on love and affection. *Neuroscience* 201 114–124 10.1016/j.neuroscience.2011.11.017 22119059

[B35] Devue C. Collette F. Balteau E. Degueldre C. Luxen A. Maquet P. (2007). Here I am: the cortical correlates of visual self-recognition. *Brain Res.* 1143 169–182 10.1016/j.brainres.2007.01.055 17306235

[B36] Diamond L. M. Dickenson J. A. (2012). The neuroimaging of love and desire: review and future directions. *Clin. Neuropsychiatry* 9 39–46.

[B37] Di Martino A. Scheres A. Margulies D. S. Kelly A. M. C. Uddin L. Q. Shehzad Z. (2008). Functional connectivity of human striatum: a resting state FMRI study. *Cereb. Cortex* 18 2735–2747 10.1093/cercor/bhn041 18400794

[B38] Farrer C. Frith C. D. (2002). Experiencing oneself vs another person as being the cause of an action: the neural correlates of the experience of agency. *Neuroimage* 15 596–603 10.1006/nimg.2001.1009 11848702

[B39] Fisher H. E. (1998). Lust, attraction, and attachment in mammalian reproduction. *Hum. Nat.* 9 23–52 10.1007/s12110-998-1010-5 26197356

[B40] Fisher H. E. Aron A. Brown L. L. (2006). Romantic love: a mammalian brain system for mate choice. *Philos. Trans. R. Soc. B Biol. Sci.* 361 2173–2186 10.1098/rstb.2006.1938 PMC1764845 17118931

[B41] Fisher H. E. Brown L. L. Aron A. Strong G. Mashek D. (2010). Reward, addiction, and emotion regulation systems associated with rejection in love. *J. Neurophysiol.* 104 51–60 10.1152/jn.00784.2009 20445032

[B42] Fox M. D. Raichle M. E. (2007). Spontaneous fluctuations in brain activity observed with functional magnetic resonance imaging. *Nat. Rev. Neurosci.* 8 700–711 10.1038/nrn2201 17704812

[B43] Fox M. D. Snyder A. Z. Vincent J. L. Corbetta M. Van Essen D. C. Raichle M. E. (2005). The human brain is intrinsically organized into dynamic, anticorrelated functional networks. *Proc. Natl. Acad. Sci. U.S.A.* 102 9673–9678 10.1073/pnas.0504136102 15976020 PMC1157105

[B44] Friston K. J. Frith C. D. Liddle P. F. Frackowiak R. S. J. (1993). Functional connectivity: the principal-component analysis of large (PET) data sets. *J. Cereb. Blood Flow Metab.* 13 5–14 10.1038/jcbfm.1993.4 8417010

[B45] Frith C. D. Frith U. (1999). Interacting minds–a biological basis. *Science* 286 1692–1695 10.1126/science.286.5445.1692 10576727

[B46] Gallagher H. L. Frith C. D. (2003). Functional imaging of ‘theory of mind’. *Trends Cogn. Sci. (Regul. Ed.)* 7 77–83 10.1016/S1364-6613(02)00025-6 12584026

[B47] Gonzaga G. C. Turner R. A. Keltner D. Campos B. Altemus M. (2006). Romantic love and sexual desire in close relationships. *Emotion* 6 163–179 10.1037/1528-3542.6.2.163 16768550

[B48] Greene J. Haidt J. (2002). How (and where) does moral judgment work? *Trends Cogn. Sci.* 6 517–523 10.1016/S1364-6613(02)02011-9 12475712

[B49] Greicius M. D. Flores B. H. Menon V. Glover G. H. Solvason H. B. Kenna H. (2007). Resting-state functional connectivity in major depression: abnormally increased contributions from subgenual cingulate cortex and thalamus. *Biol. Psychiatry* 62 429–437 10.1016/j.biopsych.2006.09.020 17210143 PMC2001244

[B50] Gusnard D. A. Akbudak E. Shulman G. L. Raichle M. E. (2001). Medial prefrontal cortex and self-referential mental activity: relation to a default mode of brain function. *Proc. Natl. Acad. Sci. U.S.A.* 98 4259–4264 10.1073/pnas.071043098 11259662 PMC31213

[B51] Hao X. Wang K. Li W. Yang W. Wei D. Qiu J. (2013). Individual differences in brain structure and resting brain function underlie cognitive styles: evidence from the embedded figures test. *PLoS ONE* 8:e78089 10.1371/journal.pone.0078089 PMC3862473 24348991

[B52] Hatfield E. Rapson R. L. (1993). Historical and cross-cultural perspectives on passionate love and sexual desire. *Annu. Rev. Sex Res.* 4 67–97.

[B53] Hatfield E. Sprecher S. (1986). Measuring passionate love in intimate relationships. *J. Adolesc.* 9 383–410 10.1016/S0140-1971(86)80043-4 3805440

[B54] Hazan C. Shaver P. (1987). Romantic love conceptualized as an attachment process. *J. Pers. Soc. Psychol.* 52 511–524 10.1037/0022-3514.52.3.511 3572722

[B55] Jankowiak W. R. Fischer E. F. (1992). A cross-cultural perspective on romantic love. *Ethnology* 31 149–155 10.2307/3773618

[B56] Kelly A. M. C. Uddin L. Q. Biswal B. B. Castellanos F. X. Milham M. P. (2008). Competition between functional brain networks mediates behavioral variability. *Neuroimage* 39 527–537 10.1016/j.neuroimage.2007.08.008 17919929

[B57] Kendrick K. (2000). Oxytocin, motherhood and bonding. *Exp. Physiol.* 85 111S–124S 10.1111/j.1469-445X.2000.tb00014.x 10795913

[B58] Knutson B. Taylor J. Kaufman M. Peterson R. Glover G. (2005). Distributed neural representation of expected value. *J. Neurosci.* 25 4806–4812 10.1523/JNEUROSCI.0642-05.2005 15888656 PMC6724773

[B59] Knutson B. Wimmer G. E. (2007). Splitting the difference. *Ann. N. Y. Acad. Sci.* 1104 54–69 10.1196/annals.1390.020 17416922

[B60] Koshino H. Kana R. K. Keller T. A. Cherkassky V. L. Minshew N. J. Just M. A. (2008). fMRI investigation of working memory for faces in autism: visual coding and underconnectivity with frontal areas. *Cereb. Cortex* 18 289–300 10.1093/cercor/bhm054 17517680 PMC4500154

[B61] Lane R. D. Fink G. R. Chau P. M.-L. Dolan R. J. (1997). Neural activation during selective attention to subjective emotional responses. *Neuroreport* 8 3969–3972 10.1097/00001756-199712220-00024 9462476

[B62] Lane R. D. Reiman E. M. Axelrod B. Yun L.-S. Holmes A. Schwartz G. E. (1998). Neural correlates of levels of emotional awareness: evidence of an interaction between emotion and attention in the anterior cingulate cortex. *J. Cogn. Neurosci.* 10 525–535 10.1162/089892998562924 9712681

[B63] Lauwereyns J. (2006). Voluntary control of unavoidable action. *Trends Cogn. Sci. (Regul. Ed.)* 10 47–49 10.1016/j.tics.2005.11.012 16359911

[B64] Lee M. H. Smyser C. D. Shimony J. S. (2013). Resting-state fMRI: a review of methods and clinical applications. *Am. J. Neuroradiol.* 34 1866–1872 10.3174/ajnr.A3263 22936095 PMC4035703

[B65] Liu C. Liu Y. Li W. Wang D. Jiang T. Zhang Y. C. (2011). Increased regional homogeneity of blood oxygen level-dependent signals in occipital cortex of early blind individuals. *Neuroreport* 22 190–194 10.1097/WNR.0b013e3283447c09 21304328

[B66] Liu H. Liu Z. Liang M. Hao Y. Tan L. Kuang F. (2006). Decreased regional homogeneity in schizophrenia: a resting state functional magnetic resonance imaging study. *Neuroreport* 17 19–22 10.1097/01.wnr.0000195666.22714.35 16361943

[B67] Liu J. Gao X. P. Osunde I. Li X. Zhou S. K. Zheng H. R. (2010). Increased regional homogeneity in internet addiction disorder a resting state functional magnetic resonance imaging study. *Chin. Med. J. (Engl)* 123 1904–1908. 20819576

[B68] Liu Y. Wang Z. X. (2003). Nucleus accumbens oxytocin and dopamine interact to regulate pair bond formation in female prairie voles. *Neuroscience* 121 537–544 10.1016/S0306-4522(03)00555-4 14568015

[B69] Lynall M.-E. Bassett D. S. Kerwin R. McKenna P. J. Kitzbichler M. Muller U. (2010). Functional connectivity and brain networks in schizophrenia. *J. Neurosci.* 30 9477–9487 10.1523/JNEUROSCI.0333-10.2010 20631176 PMC2914251

[B70] Maddock R. J. (1999). The retrosplenial cortex and emotion: new insights from functional neuroimaging of the human brain. *Trends Neurosci.* 22 310–316 10.1016/S0166-2236(98)01374-5 10370255

[B71] Mogenson G. J. Jones D. L. Yim C. Y. (1980). From motivation to action: functional interface between the limbic system and the motor system. *Prog. Neurobiol.* 14 69–97 10.1016/0301-0082(80)90018-0 6999537

[B72] Moll J. de Oliveira-Souza R. Bramati I. E. Grafman J. (2002). Functional networks in emotional moral and nonmoral social judgments. *Neuroimage* 16 696–703 10.1006/nimg.2002.1118 12169253

[B73] Ortigue S. Bianchi-Demicheli F. Hamilton A. F. C. Grafton S. T. (2007). The neural basis of love as a subliminal prime: an event-related functional magnetic resonance imaging study. *J. Cogn. Neurosci.* 19 1218–1230 10.1162/jocn.2007.19.7.1218 17583996

[B74] Ortigue S. Bianchi-Demicheli F. Patel N. Frum C. Lewis J. W. (2010). Neuroimaging of love: fMRI meta-analysis evidence toward new perspectives in sexual medicine. *J. Sex. Med.* 7 3541–3552 10.1111/j.1743-6109.2010.01999.x 20807326

[B75] Paakki J.-J. Rahko J. Long X. Moilanen I. Tervonen O. Nikkinen J. (2010). Alterations in regional homogeneity of resting-state brain activity in autism spectrum disorders. *Brain Res.* 1321 169–179 10.1016/j.brainres.2009.12.081 20053346

[B76] Paulus M. P. Stein M. B. (2006). An insular view of anxiety. *Biol. Psychiatry* 60 383–387 10.1016/j.biopsych.2006.03.042 16780813

[B77] Peele S. Brodsky A. (1975). *Love and Addiction.* Oxford: Taplinger press.

[B78] Preuschoff K. Quartz S. R. Bossaerts P. (2008). Human insula activation reflects risk prediction errors as well as risk. *J. Neurosci.* 28 2745–2752 10.1523/JNEUROSCI.4286-07.2008 18337404 PMC6670675

[B79] Raichle M. E. MacLeod A. M. Snyder A. Z. Powers W. J. Gusnard D. A. Shulman G. L. (2001). A default mode of brain function. *Proc. Natl. Acad. Sci. U.S.A.* 98 676–682 10.1073/pnas.98.2.676 11209064 PMC14647

[B80] Reynolds S. M. Zahm D. S. (2005). Specificity in the projections of prefrontal and insular cortex to ventral striatopallidum and the extended amygdala. *J. Neurosci.* 25 11757–11767 10.1523/JNEUROSCI.3432-05.2005 16354934 PMC6726011

[B81] Rilling J. K. DeMarco A. C. Hackett P. D. Thompson R. Ditzen B. Patel R. (2012). Effects of intranasal oxytocin and vasopressin on cooperative behavior and associated brain activity in men. *Psychoneuroendocrinology* 37 447–461 10.1016/j.psyneuen.2011.07.013 21840129 PMC3251702

[B82] Sabatinelli D. Bradley M. M. Lang P. J. Costa V. D. Versace F. (2007). Pleasure rather than salience activates human nucleus accumbens and medial prefrontal cortex. *J. Neurophysiol.* 98 1374–1379 10.1152/jn.00230.2007 17596422

[B83] Seymour B. O’Doherty J. P. Dayan P. Koltzenburg M. Jones A. K. Dolan R. J. (2004). Temporal difference models describe higher-order learning in humans. *Nature* 429 664–667 10.1038/nature02581 15190354

[B84] Shukla D. K. Keehn B. Müller R. A. (2010). Regional homogeneity of fMRI time series in autism spectrum disorders. *Neurosci. Lett.* 476 46–51 10.1016/j.neulet.2010.03.080 20381584 PMC2879288

[B85] Song X. W. Dong Z. Y. Long X. Y. Li S. F. Zuo X. N. Zhu C. Z. (2010). REST: a toolkit for resting-state functional magnetic resonance imaging data processing. *PLoS ONE* 6:e25031 10.1371/journal.pone.0025031 PMC3176805 21949842

[B86] Sternberg R. J. (1986). A triangular theory of love. *Psychol. Rev.* 93 119–135 10.1037/0033-295X.93.2.119

[B87] Stoffers D. Bosboom J. L. W. Deijen J. B. Wolters E. C. Stam C. J. Berendse H. W. (2008). Increased cortico-cortical functional connectivity in early-stage Parkinson’s disease: an MEG study. *Neuroimage* 41 212–222 10.1016/j.neuroimage.2008.02.027 18395468

[B88] Sue Carter C. Courtney D. A. Getz L. L. (1995). Physiological substrates of mammalian monogamy: the prairie vole model. *Neurosci. Biobehav. Rev.* 19 303–314 10.1016/0149-7634(94)00070-H 7630584

[B89] Tarlaci S. (2012). The brain in love: has neuroscience stolen the secret of love? *NeuroQuantology* 10 744–753 10.14704/nq.2012.10.4.581

[B90] Tsakiris M. Hesse M. D. Boy C. Haggard P. Fink G. R. (2007). Neural signatures of body ownership: a sensory network for bodily self-consciousness. *Cereb. Cortex* 17 2235–2244 10.1093/cercor/bhl131 17138596

[B91] Van Den Heuvel M. P. Hulshoff Pol H. E. (2010). Exploring the brain network: a review on resting-state fMRI functional connectivity. *Eur. Neuropsychopharmacol.* 20 519–534 10.1016/j.euroneuro.2010.03.008 20471808

[B92] Van Rooy D. L. Viswesvaran C. Pluta P. (2005). An evaluation of construct validity: what is this thing called emotional intelligence? *Hum. Perform.* 18 445–462 10.1207/s15327043hup1804_9

[B93] Waldherr M. Neumann I. D. (2007). Centrally released oxytocin mediates mating-induced anxiolysis in male rats. *Proc. Natl. Acad. Sci. U.S.A.* 104 16681–16684 10.1073/pnas.0705860104 17925443 PMC2034242

[B94] Wang Z. Hulihan T. J. Insel T. R. (1997). Sexual and social experience is associated with different patterns of behavior and neural activation in male prairie voles. *Brain Res.* 767 321–332 10.1016/S0006-8993(97)00617-3 9367264

[B95] Winslow J. T. Hastings N. Carter C. S. Harbaugh C. R. Insel T. R. (1993). A role for central vasopressin in pair bonding in monogamous prairie voles. *Nature* 365 545–548 10.1038/365545a0 8413608

[B96] Winston J. S. Strange B. A. O’Doherty J. Dolan R. J. (2002). Automatic and intentional brain responses during evaluation of trustworthiness of faces. *Nat. Neurosci.* 5 277–283 10.1038/nn816 11850635

[B97] Wu T. Long X. Zang Y. Wang L. Hallett M. Li K. (2009). Regional homogeneity changes in patients with Parkinson’s disease. *Hum. Brain Mapp.* 30 1502–1510 10.1002/hbm.20622 18649351 PMC6871162

[B98] Xu X. Aron A. Brown L. Cao G. Feng T. Weng X. (2011). Reward and motivation systems: a brain mapping study of early-stage intense romantic love in Chinese participants. *Hum. Brain Mapp.* 32 249–257 10.1002/hbm.21017 21229613 PMC6870433

[B99] Yao Z. Wang L. Lu Q. Liu H. Teng G. (2009). Regional homogeneity in depression and its relationship with separate depressive symptom clusters: a resting-state fMRI study. *J. Affect. Disord.* 115 430–438 10.1016/j.jad.2008.10.013 19007997

[B100] Yin J. Zhang J. X. Xie J. Zou Z. Huang X. (2013). Gender differences in perception of romance in Chinese college students. *PLoS ONE* 8:e76294 10.1371/journal.pone.0076294 PMC3797815 24146853

[B101] Zang Y. Jiang T. Lu Y. He Y. Tian L. (2004). Regional homogeneity approach to fMRI data analysis. *Neuroimage* 22 394–400 10.1016/j.neuroimage.2003.12.030 15110032

[B102] Zeki S. (2007). The neurobiology of love. *FEBS Lett.* 581 2575–2579 10.1016/j.febslet.2007.03.094 17531984

[B103] Zhang Z. Liu Y. Jiang T. Zhou B. An N. Dai H. (2012). Altered spontaneous activity in Alzheimer’s disease and mild cognitive impairment revealed by regional homogeneity. *Neuroimage* 59 1429–1440 10.1016/j.neuroimage.2011.08.049 21907292

